# Tea cultivar classification and biochemical parameter estimation from hyperspectral imagery obtained by UAV

**DOI:** 10.7717/peerj.4858

**Published:** 2018-05-28

**Authors:** Yexin Tu, Meng Bian, Yinkang Wan, Teng Fei

**Affiliations:** 1 School of Resource and Environmental Science, Wuhan University, Wuhan, China; 2 School of Remote Sensing and Information Engineering, Wuhan University, Wuhan, China; 3 Suzhou Institute, Wuhan University, Suzhou, China

**Keywords:** Hyperspectral remote sensing, Unmanned aerial vehicle, Cultivar classification, Biochemical parameter estimation, Tea quality

## Abstract

It is generally feasible to classify different species of vegetation based on remotely sensed images, but identification of different sub-species or even cultivars is uncommon. Tea trees (*Camellia sinensis* L.) have been proven to show great differences in taste and quality between cultivars. We hypothesize that hyperspectral remote sensing would make it possibly to classify cultivars of plants and even to estimate their taste-related biochemical components. In this study, hyperspectral data of the canopies of tea trees were collected by hyperspectral camera mounted on an unmanned aerial vehicle (UAV). Tea cultivars were classified according to the spectral characteristics of the tea canopies. Furthermore, two major components influencing the taste of tea, tea polyphenols (TP) and amino acids (AA), were predicted. The results showed that the overall accuracy of tea cultivar classification achieved by support vector machine is higher than 95% with proper spectral pre-processing method. The best results to predict the TP and AA were achieved by partial least squares regression with standard normal variant normalized spectra, and the ratio of TP to AA—which is one proven index for tea taste—achieved the highest accuracy (*R*_CV_ = 0.66, RMSE_CV_ = 13.27) followed by AA (*R*_CV_ = 0.62, RMSE_CV_ = 1.16) and TP (*R*_CV_ = 0.58, RMSE_CV_ = 10.01). The results indicated that classification of tea cultivars using the hyperspectral remote sensing from UAV was successful, and there is a potential to map the taste-related chemical components in tea plantations from UAV platform; however, further exploration is needed to increase the accuracy.

## Introduction

Tea (*Camellia sinensis* L.) is the second most frequently consumed beverage in the world after water (Macfarlane & Macfarlane, 2004). As a beverage, the tea’s quality decides its market value (Gallaher et al., 2006; Hilton & Palmer-Jones, 1975). Traditionally, trained sensory tasters can evaluate the tea’s quality through a series of taste indicators such as sweetness, acidity, and aroma, but sensory evaluation is unavoidably expensive and subjective. Research has revealed that tea quality is largely determined by its concentration of leaf biochemical components (Dutta, Stein & Bhagat, 2011; Yamamoto et al., 1997; Atoui et al., 2005) which are highly depended on the cultivar of tea and its growing condition (Harbowy et al., 1997; Ercisli et al., 2008; Bhatia & Ullah, 1965).

Remote sensing, especially hyperspectral remote sensing, makes it possible to classify species of plants and even to estimate their biochemical parameters, because hyperspectral data contain a great number of narrow spectral channels that can detect subtle changes in narrow absorption features (Curran, 1989). This technique has been widely applied to estimate biochemical parameters in plants, such as nitrogen (Hansen & Schjoerring, 2003; He et al., 2016; Singh et al., 2015), phosphorus (Sibanda et al., 2015; Zhou et al., 2015; Ramoelo et al., 2013), and chlorophyll (Wang, Pu & Sun, 2016; Zou et al., 2015). With regard to tea, tea polyphenols (TP) and amino acids (AA) are generally regarded as the most two influential components affecting the tea’s quality and taste (Chen et al., 2008; Potter, 2012). The ratio of TP and AA (P/A) is calculated by TP divided by AA, P/A can determine the quality of the tea to a great extent. When TP is set, the lower the P/A is, the better the quality of the tea (Wei, 2011; Bian, 2010). Although estimation of TP and AA by spectroscopy has been studied (Bian et al., 2013; Dutta et al., 2015), most of the studies are based on tea leaves in the laboratory or point measurement in the field (Bian et al., 2013; Dutta et al., 2015). Compared with point measurement, airborne and spaceborne hyperspectral data have a broader field of view and can capture the spatial characteristics and pattern of the target vegetation. However, hyperspectral instruments including airborne visible infrared imaging spectrometer, hyperspectral mapper, compact airborne spectrographic imager, Hyperion, and Tiangong-1 (TG-1) are often accompanied by a limited spatial resolution (>20 m) or relatively high cost. Because tea trees are often planted in rows with intervals of 0.5 m between rows in a tea plantation (Wang, 2000; Wen, 2006) and the spatial resolution of airborne and spaceborne instruments is much coarser than the interval of tea trees, mixed pixels with spectral information of both tea canopy and soil background are inevitably included, which will in turn decrease the accuracy of classification and the quality of estimation (Düzgün & Demirel, 2011). The existing researches at remote sensing scale only separate tea plantations from other vegetation, and have not evaluated the taste quality of tea, nor divided plants into different cultivars.

This study attempted to distinguish tea cultivars from hyperspectral images collected by unmanned aerial vehicle (UAV) and to map the biochemical concentration in the tea canopy from near ground height at the same time. The ability of a UAV to adjust its flying altitude, thus controlling the pixel size, enables the ability to obtain pure pixels of tea tree canopies and avoid the problem of mixed pixels. With the imaging hyperspectral data collected by the UAV, we can obtain and analyze the spatial distribution of the canopy biochemical parameters of *C. sinensis*.

## Materials and Methods

### Study area

**Figure 1:**
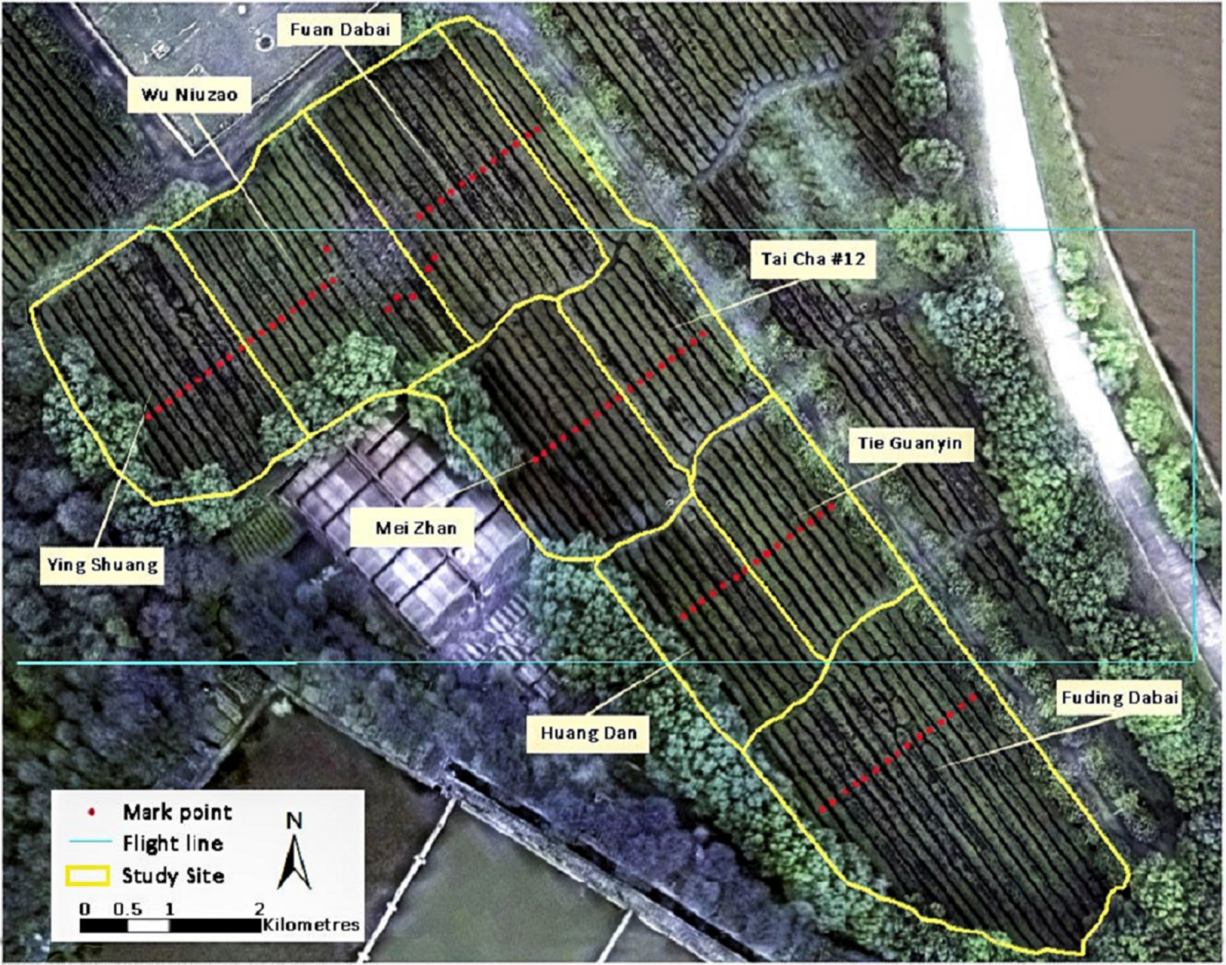
Visible RGB ortho-mosaic image of the study site on March 23, 2016, acquired using the Cubert UHD185 camera.

The study site is located in the Southlake Tea Plantation (latitude 30°28′N, longitude 114°21′E) on the eastern Jianghan Plain, Wuhan, China. The 30-year (1986–2016) average annual precipitation and sunlight are 1,000 mm and 1,850 h, respectively (China Meteorological Administration). An experimental field containing eight tea cultivars, namely Fuding Dabai, Tai Cha 12#, Huang Dan, Mei Zhan, Tie Guanyin, Wu Niuzao, Ying Shuang, and Fuan Dabai, was selected as the study area (Fig. 1).

### Hyperspectral data

**Figure 2:**
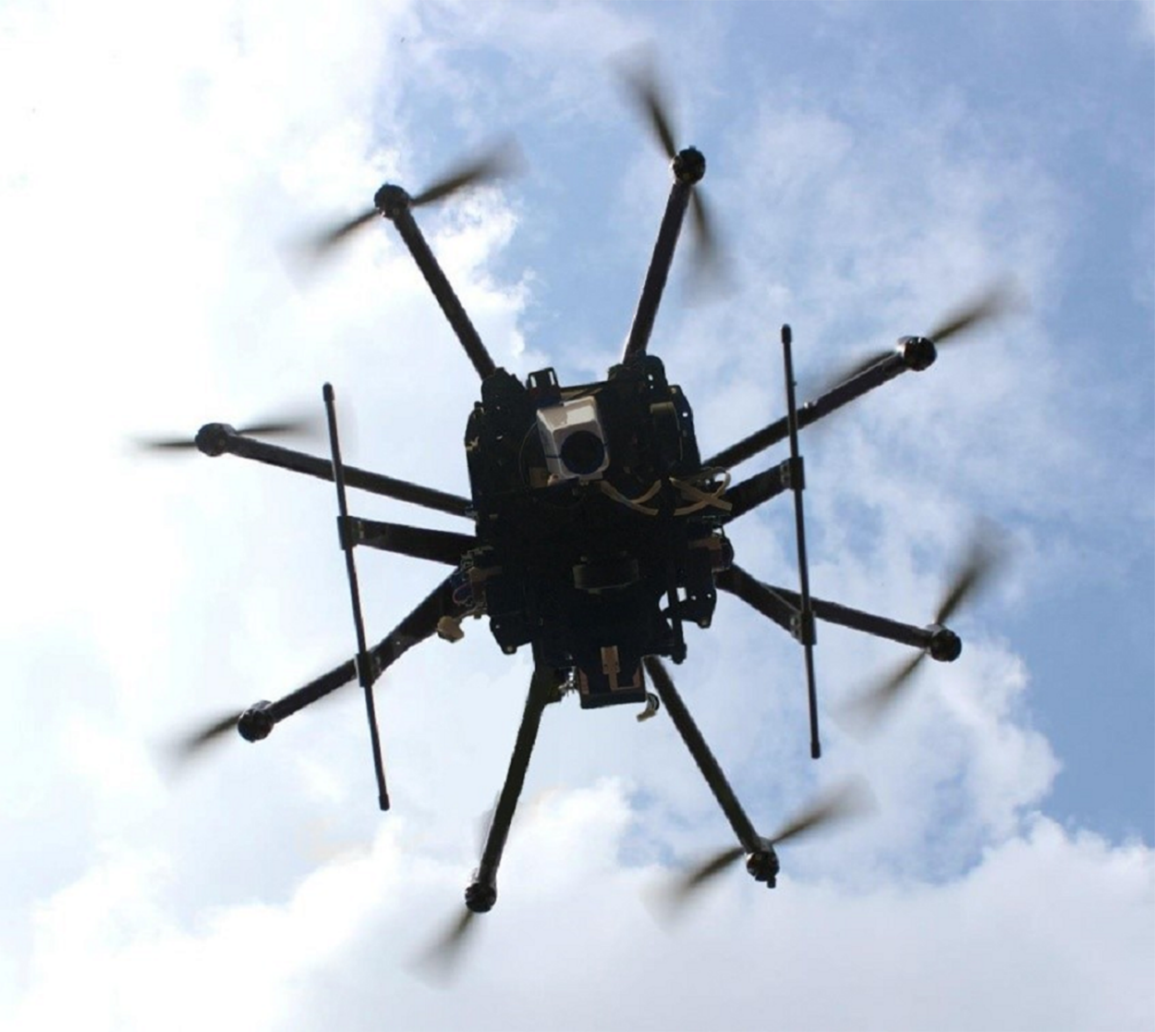
The Dji-S1000+® UAV equipped with the Cubert® mapping system. Photo by Teng Fei.

Hyperspectral images were acquired using an octocopter UAV (S1000+; DJI, Inc., Shenzhen, China) equipped with a Cubert UHD185 hyperspectral imager, (Cubert, Inc., Ulm, Germany) (Fig. 2). The imager consists of a Sony hyperspectral camera (ICX285, Sony, Inc., Tokyo, Japan), a mini-PC server, a battery power system, and the Cubert suite software.

The hyperspectral camera provided hyperspectral images in the visible and near infrared wave range. The spectral resolution of the camera was 8 nm (@532 nm) and the spectral sampling interval was 4 nm, resulting in 138 spectral channels over wavelength of 450–998 nm for each pixel. Moreover, three bands at wavelengths of 450, 550, and 650 nm share a spatial resolution of 1,000 × 1,000 pixels per frame. The other 135 bands have a spatial resolution of 50 × 50 pixels per frame. These bands were later fused to give 1,000 × 1,000 pixels through a multi-resolution image fusion algorithm embedded in the software accompanying the UHD camera.

A flight with the UAV mapping system was carried out between 10:30 and 12:00 a.m. under clear sky conditions on March 23, 2016. Before the flight, two flight lines were designed and ground control points were set over rows of tea trees to mark sampling sites for foliage collection (Fig. 1). The sensor was fixed so that the imagery was collected at nadir to enable collection of ortho-images. As the camera’s field of view angle was 29.4°, the flight height was set to 100 m, which resulted in each image frame covering an area of 54 × 54 m, with a nadir on-the-ground spatial resolution of 0.054 m. For stability of camera, the errors within the actual flight height and set height was not more than 0.1 m. The exposure time of the camera was set to 5 ms and the ISO was set to 100; therefore, the ground targets could be captured clearly while the reference of white panel in the image were not overexposed. The forward overlap and the side overlap of any two adjacent images were more than 65% and 40% respectively, and therefore most of the ground pixels were covered by at least two images.

### Foliage concentration measurement

Tea foliar chemistries of 85 samples were measured on the same day as the flight. Acquired samples were sent to a wet chemistry laboratory to measure the concentration of TP and AA. These samples were collected from 85 locations marked in Fig. 1. At each sampling point, 60–70 leaves from the canopy were collected to meet the minimum requirement with regard to foliar quantity for laboratory measurements. Statistical descriptions of TP, AA, and P/A in the study site are shown in Table 1.

**Table 1:** Biochemical components (%) of 85 foliage samples.

	Min	Max	Mean	SD
TP	46.5	110.9	85.6	1.2
AA	6.8	14.6	9.9	0.1
P/A	44.6	143.7	88.5	1.7

**Note:**

Min, minimum; Max, maximum; Mean, mean value; SD, standard deviation.

All leaf samples from the canopy were dried for 1 h at 80 °C and then ground into fine tea powder. The concentration of TP was measured by the Folin–Ciocalteu colorimetry method (Singleton, Orthofer & Lamuela-Raventos, 1999; Li & Wang, 2009; Büyükbalci & El, 2008; Schulz et al., 1999; Soultani et al., 2014; Iwasa & Torii, 1962). The AA concentration was measured using the ninhydrin colorimetry method (Lee & Takahashi, 1966; Rosen, 1957).

### Image pre-processing

Pre-processing was subsequently applied to the acquired hyperspectral images. The pre-processing was composed by the following steps:

Radiometric calibration and image selection: in this step, raw radiance spectra were converted into reflectance spectra. The UAV hyperspectral image was radiometrically corrected with reference measurements on a white board and dark measurements by covering the black plastic lens cap. The dark measurements were subtracted from the reference measurements and the actual measured values. The white board was measured twice before and after the flight. If the difference between the two measurements was larger than 5%, which means the light condition during the flight was changed significantly, the data collected during the flight was abandoned. This procedure allows us to make sure that the radiance collected are correctly translated to reflectance during the image acquisition process. After that, blurred and redundant images were discarded on the basis of visual interpretation, leaving 12 clear images covering the whole study area.

Image Fusion: due to the different spatial resolutions in different bands, image fusion was applied using the Pan-Sharpening algorithm integrated in the Cube-Pilot software (Cubert GmbH, 2014). After performing this image fusion technique, 12 image cubes with 1,000 × 1,000 pixels and 138 spectra ranging from 400–950 nm in each cube were available.

Mosaic and crop: Fused images were processed (images were aligned, a dense point cloud was built, and a mesh was built) and combined with a larger ortho-mosaic image using Agisoft PhotoScan (v1.2.4; Agisoft, Inc., St. Petersburg, Russia) (Agisoft, 2016). The large ortho-mosaic image was cropped to the study region in Envi (5.1; Harris Geospatial Solutions, Inc., Broomfield, CO, USA).

Tea canopy extraction: Although the average leave area index (LAI) of tea plants is relatively high (LAI > 3). There is still a possibility that the background soil spectra mix with the spectra of tea canopy. In order to distinguish tea plants from the soil and dry branches in the hyperspectral images, the optimized soil-adjusted vegetation index (OSAVI) was utilized as an effective index for extracting the plant canopy from the soil background. OSAVI is a soil-adjusted vegetation index optimized for agricultural monitoring, in Eq. (1).

(1) $${\rm{OSAVI}} = {{\left( {1 + {\rm{Coe}}} \right){\rm{\,*\,}}\left( {{R_{800}} - {R_{670}}} \right)} \over {\left( {{R_{800}} + {R_{670}} + {\rm{Coe}}} \right)}}$$

Rondeau (Rondeaux, Steven & Baret, 1996) summarized the formulation of OSAVI and noted that the proper value of Coe is 0.16. R800 and R670 represent the reflectance at wavelengths of 800 and 670 nm. In this study, all pixels with OSAVI larger than 0.65 were considered as tea canopy.

Dimensionality reduction: hyperspectral information is often affected by the “Hughes phenomenon,” where the classification or prediction accuracy is reduced due to the vast spectral dimension (Hughes, 1968). To avoid this effect, the raw spectra were compressed into a lower dimension by three to-be-determined preprocessing methods: minimum noise fraction (MNF) transformation, principal component analysis (PCA) and independent component analysis (ICA). These methods can decompose the spectra into statistical independent components.

### Tea cultivar classification

Supervised classifications were applied in this research. For each tea cultivar, 10–15 regions of interest were used to extract the spectral signatures for classification.

A total of four classification approaches, including both statistical model and machine learning based methods, were compared to find the possibility of tea cultivar classification and to explore the optimal methods. A total of four selected methods were maximum likelihood classification (MLC), minimum distance classification (MDC), artificial neural network (ANN) classification, and support vector machine (SVM) classification.

### Validation of tea cultivar classification

To validate the classification accuracy, tea cultivar types at 500 randomly distributed points in the image were identified during a fieldwork. The confusion matrix (Stehman, 1997) was then calculated by comparing the ground truth data with the classification results. The overall accuracy (OA) and Kappa coefficient (Kappa) in the confusion matrix indicate the percentage of correctly classified pixels and the effectiveness of the overall classification. OA and Kappa are described by Eqs. (2) and (3) as follows:

(2) $${\rm{OA}} = {{\mathop \sum \limits_{i = 1}^T {X_{ii}}} \over n}$$

(3) $${\rm{Kappa}} = {{n * \sum\nolimits_{i = 1}^T {Xii - \sum\nolimits_{i = 1}^T {\sum\nolimits_{j = 1}^T {Xij} } } } \over {{n^2} - \sum\nolimits_{i = 1}^T {\sum\nolimits_{j = 1}^T {Xij} } }}$$

In Eqs. (2) and (3), *T* is the number of classes; *Xii* represent the correctly classified pixels in class *i*; *Xij* represent the incorrectly classified pixels in class *i*; and *n* represents the number of pixels participating in the classification.

### Leaf biochemistry prediction from canopy

In order to establish a robust biochemical prediction model, several pre-processing methods were applied to the spectra, including the Wavelet De-noising method, continuum removal, standard normal variant (SNV), first derivative, and second derivative.

The spectral bands centered at 450 nm and between 954 and 998 nm were excluded, because these bands were considered noisy with a low SNR (36). Thus, spectra between 450 and 954 nm were left for modeling.

Partial least squares regression (PLSR) was performed to establish the relationship between the reflectance and the foliar chemistry of tea leaves, because it has proven accuracy and effectiveness when solving small samples and high dimensional regression problems (Shi et al., 2014; Liu et al., 2014). Besides, the predictive ability and stability of the PLSR model are often based on the selection of a number of factors (Asyraf & Afthanorhan, 2013).

Artificial neural network has shown good performances in solving non-linear regression and has high error tolerance (Keiner & Yan, 1998). To tackle the possible nonlinearity in the biochemical parameter estimation, a back-propagation ANN (BP-ANN) was tried with the same prepressing methods, and the best results were obtained by using the PLS method. The modeling work was coded with Matlab® (R2014a; MathWorks, Inc., Natick, MA, USA).

The leave-one-out cross-validation method was employed to estimate the accuracy of the model to prevent over-fitting during training (Kohavi, 1995). All samples were divided into several parts: 84 samples were used as the calibration data set for regression and one sample was used as the validation data set to validate the results. The regression did not stop until every sample was assigned to the validation set once. The models were evaluated by comparing the average correlation coefficient of cross-validation (*R*_CV_) and the averaged root mean squared error of cross-validation (RMSE_CV_). *R*_CV_ and RMSE_CV_ measure how well the model fits the data: the larger the value of *R*_CV_ and the smaller the value of RMSE_CV_, the greater the precision and accuracy of the model. *R*_CV_ and RMSE_CV_ are computed by Eqs. (4) and (5),

(4) $${R_{{\rm{cv}}}} = {{\mathop \sum \nolimits_{i = 1}^N\left( {{{\hat y}_i} - {\rm{mean}}\left( {{{\hat y}_i}} \right)} \right)\left( {{y_i} - {\rm{mean}}\left( {{y_i}} \right)} \right)} \over {\sqrt {\mathop \sum \nolimits_i^N{{\left[ {{{\hat y}_i} - {\rm{mean}}\left( {{{\hat y}_i}} \right)} \right]}^2\,*\,} \left[ {{y_i} - {\rm{mean}}\left( {{y_i}} \right)} \right]^2}}}$$

(5) $${\rm{RMS}}{{\rm{E}}_{{\rm{CV}}}} = \sqrt {{1 \over N}*\mathop \sum \limits_{i = 1}^N {{\left( {{{\hat y}_i} - {y_i}} \right)}^2}} $$

where *N* is the number of samples, $\hat {{y_i}}$ and *y_i_* and are the predicted and measured values of biochemical components of each sample. The mean ($\hat {{y_i}}$) and mean (*y_i_*) represent the average values of $\hat {y{_i}}$ and *y_i_*.

## Results

### Classification of tea cultivars

**Figure 3:**
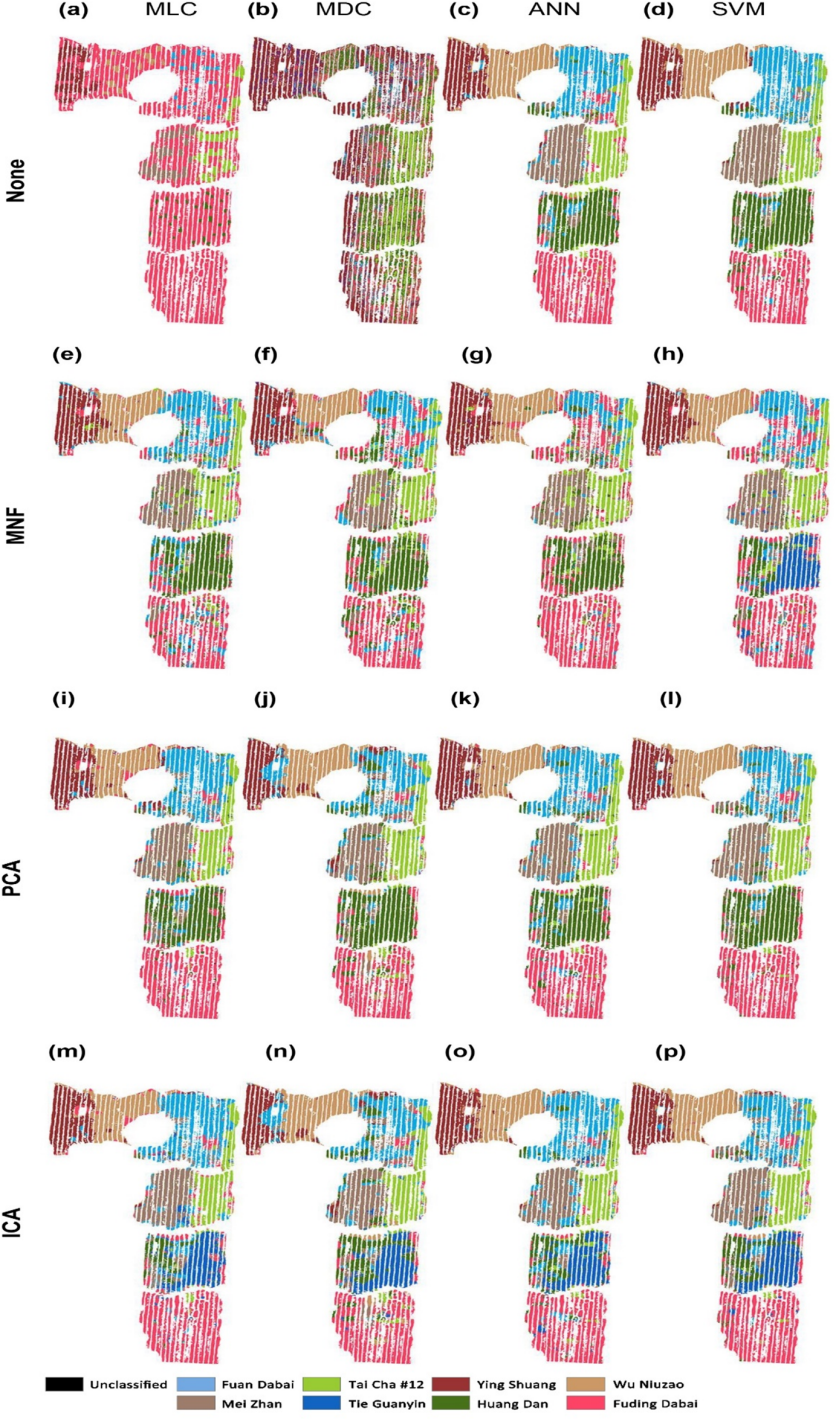
Classification of tea cultivars in the study region, with image pre-processing and classification method combinations of: (A) None+MLC (B) None+MDC (C) None+ANN (D) None+SVM (E) MNF+MLC (F) MNF+MDC (G) MNF+ANN (H) MNF+SVM (I) PCA+MLC (J) PCA+MDC (K) PCA+ANN (L) PCA+SVM (M) ICA+MLC (N) ICA+MDC (O) ICA+ANN (P) ICA+SVM.

The tea cultivar classification maps were generated from the hyperspectral data by using full wave bands with different models of MLC, MDC, ANN, and SVM, as shown in Figs. 3A–3D. The results obtained by employing the dimension reduction methods of MNF transformation (e–h), PCA (i–l), and ICA (m–p) are shown in the second to the fourth rows in the same figure.

The confusion matrix is calculated based on the classification results against the 500 validation points. The OA and Kappa accuracy evaluation indices are shown in Table 2. With the full-band spectra, a relatively low classification accuracy is achieved by using MLC and MDC, which result in OAs of 48.4 and 35.2%, and values of the Kappa coefficient of 41.5% and 26.0%, respectively (Table 2). MLC and MDC do not clearly distinguish between the eight cultivars of tea. The MLC algorithm classifies almost all of the tea plants as Fuding Dabai cultivar, and MDC classifies most of them as Ying Shuang. ANN and SVM give better classification results than MLC and MDC according to the OA. Cultivar boundaries are obvious under ANN and SVM classification, and the spatial distribution of tea cultivars is basically correct (Figs. 3C and 3D). ANN shows poor ability to identify the cultivar Huang Dan, while SVM performs better. However, it is time consuming to carry out ANN and SVM classification on the hyperspectral image cube on a PC (220 and 420 min on a 3-GHZ 24-core workstation).

**Table 2:** Accuracy evaluation of the tea cultivar classification.

Classification method	MLC (%)	MDC (%)	ANN (%)	SVM (%)
Dimensionality reduction method: None				
OA	48.4	35.2	93.2	96.2
Kappa	41.5	26.0	92.2	95.6
Dimensionality reduction method: MNF				
OA	84.0	79.8	84.8	87.6
Kappa	81.6	76.8	82.1	85.8
Dimensionality reduction method: PCA				
OA	86.8	78.2	90.2	95.2
Kappa	84.9	75.0	88.8	94.5
Dimensionality reduction method: ICA				
OA	89.4	80.4	90.4	93.8
Kappa	87.8	77.4	89.0	92.9

Minimum noise fraction analysis revealed that the top 10 bands possessed more than 90% of the information in the full-band spectra; therefore, these 10 bands were selected as the new input spectra. As shown in Figs. 3E–3G, the classification results of MLC and MDC are much better than those without MNF transformation. The values of OA and Kappa are twice those obtained without dimensionality reduction (Table 2). As shown in Table 2, worse values of OA and Kappa were obtained with the ANN and SVM methods compared to cases in which the full spectra were used. With dimension reduction of the spectra, the processing time decreased to less than 60 min when using the ANN and SVM algorithms. In brief, MNF can improve the classification accuracy dramatically in statistical models like MLC and MDC but it fails in machine learning algorithms like ANN and SVM.

Through PCA analysis, it was found that the first three principle components contained nearly 90% of the full-band information; therefore, the first three components were used for classification. With PCA, the classification results were similar to the results of MNF, especially for the statistical models such as MLC and MDC, but significantly better results were obtained by ANN and SVM classification. As only three instead of 138 bands were used, the classification efficiency is much higher than without PCA and MNF transformation and the computational expense is reasonable: less than 30 min when using the ANN and SVM algorithms.

When we performed the forward ICA transformation, 10 independent components were used to replace the full 138 bands. After ICA transformation, SVM still obtains the best classification result among the modeling methods. In general, MLC, MDC, ANN, and SVM show similar classification accuracy after PCA transformation.

Among the various classification methods, SVM classification always shows the best result, while ANN classification shows slightly poorer performance. The results of MLC and MDC are worse than those of the above machine learning algorithms. By reducing the dimension of hyperspectral data, the classification accuracy of statistical classification improves considerably, while the improvement in classification accuracy is not obvious for machine learning algorithms.

### Prediction of foliar biochemistry

The results indicate that the pre-processing methods have a significant impact on the prediction accuracy (Table 3). The best calibration results for TP, AA, and P/A were achieved by using the SNV pre-processing method (*R*_CV_ = 0.58, 0.62, 0.66 and RMSE_CV_ = 10.01, 1.16, 13.27). By using PLS regression with the SNV pre-processing method, the best estimation results were obtained.

**Table 3:** *R*_CV_ and RMSE_CV_ (g kg^−1^) of PLS regression models with multivariate pre-processing methods.

Target	Factors	TP		AA		P/A	
Preprocessing method		*R*_CV_	RMSE_CV_	*R*_CV_	RMSE_CV_	*R*_CV_	RMSE_CV_
None	7	0.57	10.04	0.50	1.31	0.60	14.16
WD	8	0.50	10.79	0.49	1.33	0.55	14.93
CR	9	0.45	11.24	0.40	1.40	0.48	15.87
SNV	7	0.58	10.01	0.62	1.16	0.66	13.27
WD+CR	9	0.49	10.77	0.35	1.46	0.42	16.37
WD+SNV	5	0.48	10.60	0.51	1.25	0.52	14.80
CR+SNV	6	0.47	12.03	0.48	2.57	0.54	14.68
WD+CR+SNV	10	0.52	10.55	0.44	1.46	0.50	15.49
CR+First+SNV	9	0.42	11.55	0.30	1.52	0.43	16.57
CR+Second+SNV	10	0.32	12.68	0.29	1.53	0.47	15.89

In order to model the potential non-linear relationship between the spectral reflectance and foliar chemistry, a BP-ANN was established with the same inputs as the PLS regression with the best pre-processing methods (SNV). The result (Table 4) shows no significant improvement when using ANN regression, besides the computational expense is much higher using ANN regression.

**Table 4:** *R*_CV_ and RMSE_CV_ (g kg^−1^) of ANN regression model with SNV pre-processing methods.

Target	TP		AA		P/A	
Hidden layer	*R*_CV_	RMSE_CV_	*R*_CV_	RMSE_CV_	*R*_CV_	RMSE_CV_
9	0.51	12.81	0.52	1.37	0.51	17.11

**Figure 4:**
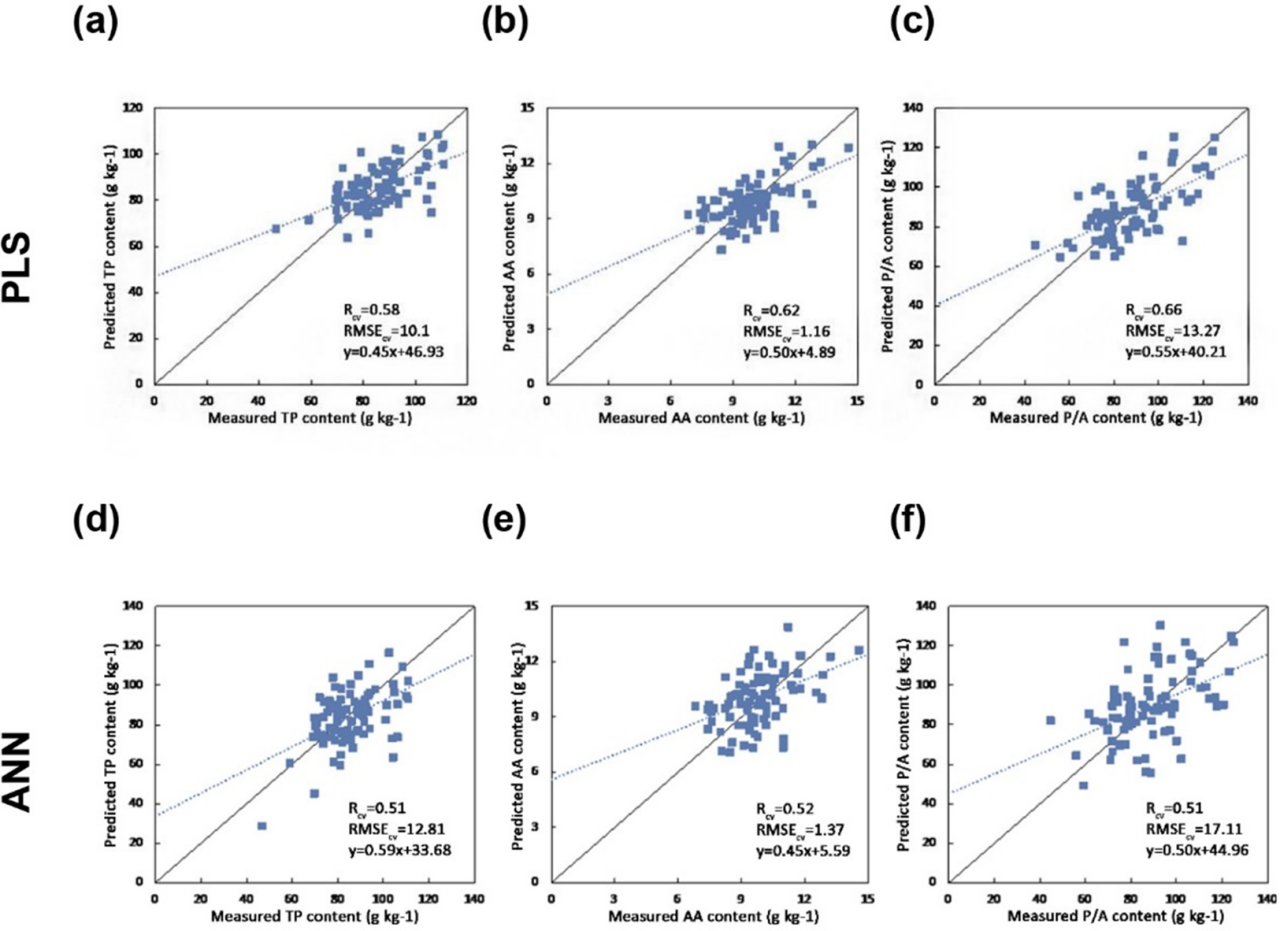
Scatter plots of the reference versus predicted foliar biochemical contents (g kg-1) using PLS and ANN regression (the solid line is the 1:1 line and the dashed line is the regression line between the predicted and measured values) (A) Predicting TP using PLS regression (B) Predicting AA using PLS regression (C) Predicting P/A using PLS regression (D) Predicting TP using ANN (E) Predicting AA using ANN (F) Predicting P/A using ANN.

Figure 4 shows the one-one plot between the predicted and measured biochemical concentrations using PLSR and ANN regression. The PLS regression obtains better results in terms of *R*_cv_ and RMSE_CV_.

## Discussion

In the field of tea plantation management, this study accomplished two tasks: to accurately classify tea plants into cultivars and to estimate the quality-related foliar biochemicals of tea plants at near ground scale. Since few reports on classifying plants into cultivars using remote sensing were found, this research seems to be the first to demonstrate the feasibility of cultivar classification with hyperspectral data collected from UAV platform.

In the prediction of foliar biochemical components, with our result it is safe to state that prediction of the biochemical components of tea plants is feasible at near ground scale. Compared with contemporary spectroscopy researches, however, our biochemical estimation results are generally less accurate than those at leaf powder scale (*R*^2^ from 0.91 to 0.99) and foliage scale (*R*^2^ from 0.54 to 0.71) (Bian, 2010; Gong, Pu & Heald, 2002; Dury & Turner, 2001). When upscaling to the near ground scale, further factors such as leaf angle, canopy structure, soil background, and atmospheric scattering and absorption weaken the SNR of spectral reflectance, as a result, the accuracy of the bio-chemical parameter reported in our study is not as high as the studies focused at powder or leave scale. However, with this sacrifice, for the first time, one can monitor the spatial distribution pattern of TP and AA in a tea plantation.

The beta coefficients (β-coefficients) in PLS are often used to determine the importance of spectral bands in PLS calibrations (Haaland & Thomas, 1988). A waveband is considered significant if the corresponding β-coefficient exceed a threshold, for example one standard deviation (±σ). We calculated the prediction models repeatedly: 85 times for each biochemical component. Wavebands that were identified as important more than 75 times (>88%) were regarded as the robust ones in this research. The important wavelengths were centered near to 454, 490, 538, and 578 nm for the prediction of TP; 470, 482, and 522 nm for AA; and 482, 502, 522, and 534 nm for P/A. We found that the wavebands from 522 to 578 nm played an important role in the three main predictions. The wavelength range was identified as the most useful wavelengths for predicting the chlorophyll a/b and the vital component nitrogen contents in other spectroscopy researches (Blackburn, 1998; Mutanga, Skidmore & Prins, 2004; Penuelas et al., 1994). Because nitrogen is also a major component of AA, when estimating free AA, some of the influential bands are close to the nitrogen absorption peaks.

As we mentioned earlier, the UHD185 sensor uses an image fusion technique to generate full resolution image cubes from hyperspectral data with resolution of 50 by 50 and RGB data with resolution of 1,000 by 1,000. This design makes assumptions about the correlation of hyperspectral bands with nearby visible wavelengths at 450, 550, and 650nm. The first three principal components from PCA analysis and the original high spatial resolution visible bands (450, 550, 650 nm) used for image fusion are compared in terms of correlation coefficients. It is quite obvious that the second principle component is highly correlated with the visible band at 650 nm, while the other two components are poorly related with all visible bands (Table 5). Not so strictly speaking, the second principle component can be seen as a proxy spectral band of red.

**Table 5:** Correlation coefficient of components and visible bands.

	PCA1	PCA2	PCA3
Red (650 nm)	0.21	0.97	0.03
Green (550 nm)	0.51	0.85	−0.05
Blue (450 nm)	0.42	0.57	0.48

**Figure 5:**
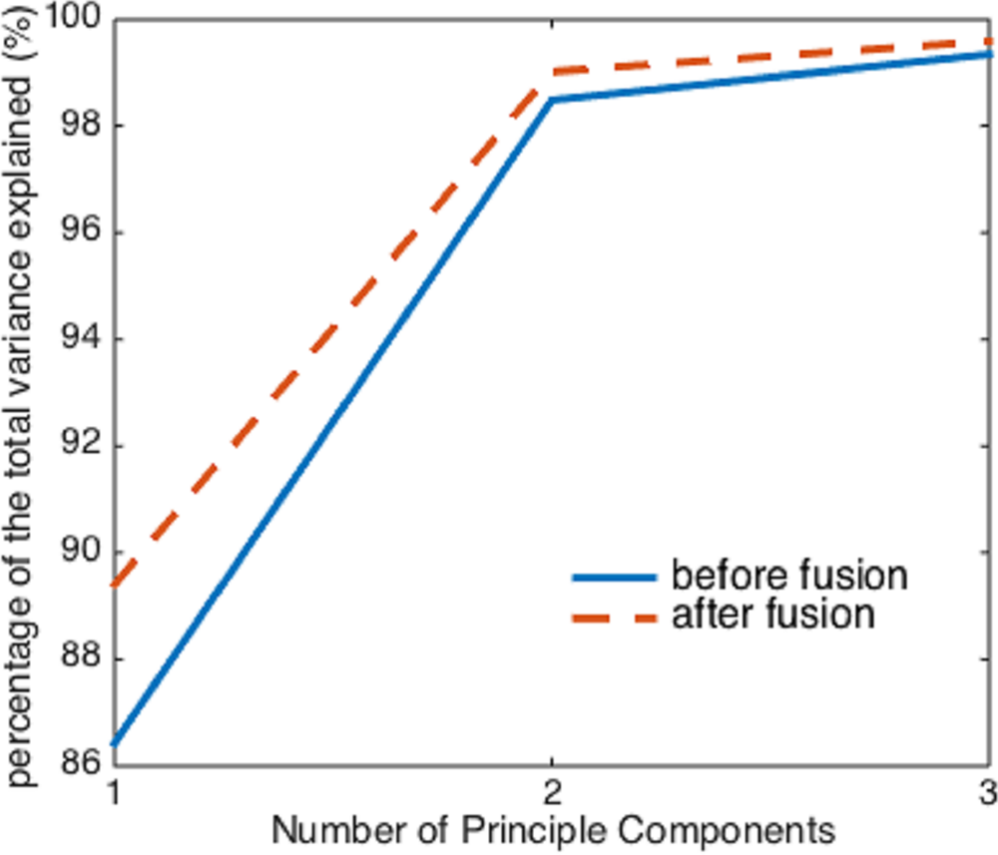
The percentage of the variance explained by the first three principal components before and after image fusion.

However, before and after the image fusion, the first three principal components of the two hyperspectral cubes can explain almost the same percentage (>99%) of the total variation in the raw spectral data, despite the image fusion process enhances the collinearity between spectra (Fig. 5). Therefore, if using more than two principal components as independent variables, it seems that the image fusion process does not have a significant impact on the effectiveness of PCA.

The research has important practical significance for tea quality evaluation and also provides a reference for tea plantation management and precision agriculture. Further researches can be performed to further explore the potential of hyperspectral images onboard a UAV, such as by making use of the information on spatial auto correlation to improve the accuracy of parameter estimation or by using images taken by UAV at different altitudes to seek the best spatial scale to estimate the canopy biochemical parameters.

## Conclusion

This study indicated that the classification of tea cultivars using the hyperspectral remote sensing from UAV can achieve high accuracy. Although there is a strong statistical significance between the sensory quality of tea and the canopy spectra, the potential to map the taste-related chemical components in tea plantations from UAV platform, however, needs further exploration to increase the accuracy.

## Supplemental Information

10.7717/peerj.4858/supp-1 Supplemental Information 1 Measured TP and AA content.

## Additional Information and Declarations

### Data Availability

The following information was supplied regarding data availability: Hyperspectral Images obtained by UAV, measured TP and AA content were used as raw data and uploaded to Figshare: https://figshare.com/articles/spectra_data_of_tea_plantation/5844801. Fei, Teng (2018): Spectra data of tea plantation. figshare. Figure. https://doi.org/10.6084/m9.figshare.5844801.v1.

## References

[ref-1] Agisoft (2016). Agisoft user manuals.

[ref-2] Asyraf M, Afthanorhan BW (2013). A comparison of partial least square structural equation modeling (pls-sem) and covariance based structural equation modeling (cb-sem) for confirmatory factor analysis. International Journal of Engineering, Science and Innovation Technologies.

[ref-3] Atoui AK, Mansouri A, Boskou G, Kefalas P (2005). Tea and herbal infusions: their antioxidant activity and phenolic profile. Food Chemistry.

[ref-4] Bhatia IS, Ullah MR (1965). Quantitative changes in the polyphenols during the processing of tea leaf and their relation to liquor characters of made tea. Journal of the Science of Food and Agriculture.

[ref-5] Bian M (2010). Using hyperspectral remote sensing of foliar chemicals to predict the quality of tea (*Camellia Sinensis*).

[ref-6] Bian M, Skidmore AK, Schlerf M, Wang T, Liu Y, Zeng R, Fei T (2013). Predicting foliar biochemistry of tea (*Camellia sinensis*) using reflectance spectra measured at powder, leaf and canopy levels. ISPRS Journal of Photogrammetry and Remote Sensing.

[ref-7] Blackburn GA (1998). Quantifying chlorophylls and caroteniods at leaf and canopy scales: an evaluation of some hyperspectral approaches. Remote Sensing of Environment.

[ref-8] Büyükbalci A, El SN (2008). Determination of in vitro antidiabetic effects, antioxidant activities and phenol contents of some herbal teas. Plant Foods for Human Nutrition.

[ref-9] Chen Q, Zhao J, Liu M, Cai J, Liu J (2008). Determination of total polyphenols content in green tea using FT-NIR spectroscopy and different PLS algorithms. Journal of Pharmaceutical and Biomedical Analysis.

[ref-10] Cubert GmbH (2014). Cubert Software and Installation Guide.

[ref-11] Curran PJ (1989). Remote sensing of foliar chemistry. Remote Sensing of Environment.

[ref-12] Dury SJ, Turner BJ (2001). Nutrient estimation of eucalypt foliage derived from hyperspectral data.

[ref-13] Dutta D, Das PK, Bhunia UK, Singh U, Singh S, Sharma JR, Dadhwal VK (2015). Retrieval of tea polyphenol at leaf level using spectral transformation and multi-variate statistical approach. International Journal of Applied Earth Observation and Geoinformation.

[ref-14] Dutta R, Stein A, Bhagat RM (2011). Integrating satellite images and spectroscopy to measuring green and black tea quality. Food Chemistry.

[ref-15] Düzgün HŞ, Demirel N (2011). Remote Sensing of the Mine Environment.

[ref-16] Ercisli S, Orhan E, Ozdemir O, Sengul M, Gungor N (2008). Seasonal variation of total phenolic, antioxidant activity, plant nutritional elements, and fatty acids in tea leaves (*Camellia sinensis* var. sinensis clone Derepazari 7) grown in Turkey. Pharmaceutical Biology.

[ref-17] Gallaher RN, Gallaher K, Marshall AJ, Marshall AC (2006). Mineral analysis of ten types of commercially available tea. Journal of Food Composition and Analysis.

[ref-18] Gong P, Pu R, Heald RC (2002). Analysis of in situ hyperspectral data for nutrient estimation of giant sequoia. International Journal of Remote Sensing.

[ref-19] Haaland DM, Thomas EV (1988). Partial least-squares methods for spectral analyses: 1. Relation to other quantitative calibration methods and the extraction of qualitative information. Analytical Chemistry.

[ref-20] Hansen PM, Schjoerring JK (2003). Reflectance measurement of canopy biomass and nitrogen status in wheat crops using normalized difference vegetation indices and partial least squares regression. Remote Sensing of Environment.

[ref-21] Harbowy ME, Balentine DA, Davies AP, Cai Y (1997). Tea chemistry. Critical Reviews in Plant Sciences.

[ref-22] He L, Song X, Feng W, Guo B-B, Zhang Y-S, Wang Y-H, Wang C-Y, Guo TC (2016). Improved remote sensing of leaf nitrogen concentration in winter wheat using multi-angular hyperspectral data. Remote Sensing of Environment.

[ref-23] Hilton PJ, Palmer-Jones RW (1975). Chemical assessment of quality in tea and its relation to the market over an extended period. Journal of the Science of Food and Agriculture.

[ref-24] Hughes GF (1968). On the mean accuracy of stastistical pattern recognizers. IEEE Transactions on Information Theory.

[ref-25] Iwasa K, Torii HA (1962). Colorimetric determination of tea tannin with ferrous tartrate. Study of Tea.

[ref-26] Keiner LE, Yan XH (1998). A neural network model for estimating sea surface chlorophyll and sediments from thematic mapper imagery. Remote Sensing of Environment.

[ref-27] Kohavi R (1995). A study of cross-validation and bootstrap for accuracy estimation and model selection.

[ref-29] Lee YP, Takahashi T (1966). An improved colorimetric determination of amino acids with the use of ninhydrin. Analytical Biochemistry.

[ref-30] Li J, Wang B (2009). Folin–Ciocalteu colorimetric determination of total polyphenols in mulberry fruits. Food Science.

[ref-31] Liu Y, Jiang Q, Fei T, Wang J, Shi T, Guo K (2014). Transferability of a visible and near-infrared model for soil organic matter estimation in riparian landscapes. Remote Sensing.

[ref-32] Macfarlane A, Macfarlane I (2004). The Empire of Tea.

[ref-33] Sibanda M, Mutanga O, Rouget M, Odindi J (2015). Exploring the potential of in situ hyperspectral data and multivariate techniques in discriminating different fertilizer treatments in grasslands. Journal of Applied Remote Sensing.

[ref-34] Mutanga O, Skidmore AK, Prins HHT (2004). Predicting in situ pasture quality in the Kruger National Park, South Africa, using continuum-removed absorption features. Remote Sensing of Environment.

[ref-35] Potter H (2012). Uncovering the secrets of tea. http://www.rsc.org/chemistryworld/2012/11/tea-health-benefits.

[ref-36] Penuelas J, Gamon JA, Fredeen AL, Merino J, Field CB (1994). Reflectance indices associated with physiological changes in nitrogen- and water-limited sunflower leaves. Remote Sensing of Environment.

[ref-37] Ramoelo A, Skidmore AK, Cho MA, Mathieu R, Heitkönig IMA, Dudeni-Tlhone N, Schlerf M, Prins HHT (2013). Non-linear partial least square regression increases the estimation accuracy of grass nitrogen and phosphorus using in situ, hyperspectral and environmental data. ISPRS Journal of Photogrammetry and Remote Sensing.

[ref-38] Rondeaux G, Steven M, Baret F (1996). Optimization of soil-adjusted vegetation indices. Remote Sensing of Environment.

[ref-39] Rosen H (1957). A modified ninhydrin colorimetric analysis for amino acids. Archives of Biochemistry and Biophysics.

[ref-40] Schulz H, Engelhardt UH, Wegent A, Drews H, Lapczynski S (1999). Application of near-infrared reflectance spectroscopy to the simultaneous prediction of alkaloids and phenolic substances in green tea leaves. Journal of Agricultural and Food Chemistry.

[ref-41] Shi T, Liu H, Wang J, Chen Y, Fei T, Wu G (2014). Monitoring arsenic contamination in agricultural soils with reflectance spectroscopy of rice plants. Environmental Science & Technology.

[ref-42] Singh A, Serbin SP, Mcneil BE, Kingdon CC, Townsend PA (2015). Imaging spectroscopy algorithms for mapping canopy foliar chemical and morphological traits and their uncertainties. Ecological Applications.

[ref-28] Singleton VL, Orthofer R, Lamuela-Raventos RM (1999). Analysis of total phenols and other oxidation substrates and antioxidants by means of Folin-Ciocalteu reagent. Methods in Enzymology.

[ref-43] Soultani G, Evageliou V, Koutelidakis AE, Kapsokefalou M, Komaitis M (2014). The effect of pectin and other constituents on the antioxidant activity of tea. Food Hydrocolloids.

[ref-44] Stehman SV (1997). Selecting and interpreting measures of thematic classification accuracy. Remote Sensing of Environment.

[ref-45] Wang G (2000). Practicing and thinking on young tea garden intercropping. Tea Communication.

[ref-46] Wang L, Pu H, Sun DW (2016). Estimation of chlorophyll-a concentration of different seasons in outdoor ponds using hyperspectral imaging. Talanta.

[ref-47] Wei C (2011). Different fertilizers influence the quality of tea.

[ref-48] Wen Z (2006). An approach for establishment of production base of organic tea. Journal of Guangxi Agriculture.

[ref-49] Yamamoto T, Juneja LR, Chu DC, Kim M (1997). Chemistry and Applications of Green Tea.

[ref-50] Zhou G, Niu C, Xu W, Yang W, Wang J, Zhao H (2015). Canopy modeling of aquatic vegetation: a radiative transfer approach. Remote Sensing of Environment.

[ref-51] Zou X, Hernández-Clemente R, Tammeorg P, Torres CL, Stoddard FL, Mäkelä P, Pellikka P, Mõttus M (2015). Retrieval of leaf chlorophyll content in field crops using narrow-band indices: effects of leaf area index and leaf mean tilt angle. International Journal of Remote Sensing.

